# Supplementary material for: Transcription leads to pervasive replisome instability in bacteria
Source: eLife. 2017 Jan 16;6:e19848. doi: 10.7554/eLife.19848 (PMC5305214; doi:10.7554/eLife.19848)
Supplement: Supplementary file 1. — DOI: http://dx.doi.org/10.7554/eLife.19848.038 [file elife-19848-supp1.pdf]

Independent-fork model

In our analysis of the focus dynamics, we have not distinguished between one and two fork replisomes and interpreted the measured rate of focus loss as the rate of single fork disassembly to generate a crude estimate of the number of conflicts per cell cycle. We will call this qualitative model the *cooperative-fork model* since it implicitly postulates that a conflict at a single fork results is loss of the replication complex at both forks. While this model is clearly a useful qualitative device for making a crude estimate of the number of conflicts per cell cycle, it is inconsistent with the stoichiometry data which demonstrates that replisomes frequently have a stoichiometry consistent with a single fork. We now briefly explore a more self-consistent model which distinguishes between one and two-fork complexes. We will assume that the replication forks move and act independently and assume that the conflict-generation and replication restart are both Poisson processes with a constant rate:

$$\text{active} \underset{k_+}{\overset{k_-}{\rightleftharpoons}} \text{stalled}, \tag{1}$$

for a single fork. Since the forks in this model act independently, we will call this model the *independent-fork model*. This more complicated model changes none of the qualitative insights from the cooperative fork model.

Model and rates. Since our experiments are sensitive only to the number of assembled fork complexes, we define the states in terms of the number of complexes in the focus: 0, 1, or 2. The rate equation describing the number of two, one and zero active complexes is therefore:

$$\begin{pmatrix} \dot{N}_2 \\ \dot{N}_1 \\ \dot{N}_0 \end{pmatrix} = \begin{pmatrix} -2k_- & k_+ & 0 \\ 2k_- & -k_+ - k_- & 2k_+ \\ 0 & k_- & -2k_+ \end{pmatrix} \begin{pmatrix} N_2 \\ N_1 \\ N_0 \end{pmatrix}, \tag{2}$$

where the factors of two in the rate equation account for the fact that either of the two forks can disassemble. We analyze the linear model in the canonical way: we find the eigenvalues and vectors.

Steady-state solution. In the current context, we only care about the steady-state solution with eigenvalue zero:

$$\boldsymbol{\psi}_0^T = \begin{pmatrix} k_+^2/k_-^2 & 2k_+/k_- & 1 \end{pmatrix}, \tag{3}$$

which determines the ratios between two and one fork replisomes is steady state:

$$R \equiv N_2(\infty)/N_1(\infty) = k_+/2k_-. \tag{4}$$

where the factor of two in the denominator is the result of the ability of either fork to transition to the stalled state. R is fit from the stoichiometry measurements and is therefore observable.

The second observable is the lifetime of a focus which is measured in the replisome dynamics experiments. As we demonstrate in the next section, the average lifetime is:

$$\bar{t} = \frac{4k_-^2 + 5k_-k_+ + k_+^2}{2k_-^2(2k_- + k_+)}, \tag{5}$$

as a function of the independent-fork model rates $k_\pm$. We will discuss the effective rate of focus loss $k_{\text{eff}} \equiv \bar{t}^{-1}$. An intuitive limit for k_{eff} is the large k_+, where the effective rate of focus loss is:

$$k_{\text{eff}} \approx \frac{2k_-^2}{k_+}. \tag{6}$$

The rate has a squared dependence on k_- and inverse dependence on k_+ since two successive conflict transitions are required without restart to lose a focus. This limit is *not* relevant in the current context since $k_- \approx k_+$.

Technical details: **The first-passage-time calculation.** To analyze the lifetime, we will solve the first passage time problem using the canonical method: We make the zero-focus state absorbing, preventing transitions from 0 to 1 forks. The average lifetime for system is defined:

$$\bar{t} \equiv \int_0^\infty \mathrm{d}t \; t \; p_{1\to 0}(t), \tag{7}$$

where $p_{1\to 0}(t)$ is the probability density of transitions from the one-fork to the zero fork state. Number conservation implies:

$$p_{1\to 0}(t) = -\frac{\mathrm{d}}{\mathrm{d}t} \begin{pmatrix} 1 & 1 \end{pmatrix} \begin{pmatrix} P_2(t) \\ P_1(t) \end{pmatrix}, \tag{8}$$

where P_i is the probability of occupancy for the i fork state. To perform this calculation, we project onto the N_2-N_1 plane:

$$\boldsymbol{K}_* = \begin{pmatrix} -2k_- & k_+ \\ 2k_- & -k_+ - k_- \end{pmatrix}. \tag{9}$$

We integrate Eqn. 7 by parts. We then use the solution to the matrix linear differential equation for the probabilities, given the initial conditions:

$$\begin{pmatrix} P_2(t) \\ P_1(t) \end{pmatrix} = \exp\left[\boldsymbol{K}_* t\right] \begin{pmatrix} P_2(0) \\ P_1(0) \end{pmatrix}. \tag{10}$$

The resulting integral can be easily evaluated:

$$\bar{t} = \int_0^\infty \mathrm{d}t \; \begin{pmatrix} 1 & 1 \end{pmatrix} \exp\left[\boldsymbol{K}_* t\right] \begin{pmatrix} P_2(0) \\ P_1(0) \end{pmatrix}, \tag{11}$$

$$= -\begin{pmatrix} 1 & 1 \end{pmatrix} \boldsymbol{K}_*^{-1} \begin{pmatrix} P_2(0) \\ P_1(0) \end{pmatrix}. \tag{12}$$

The inverse rate matrix $\boldsymbol{K}_*^{-1}$ can be computed using the well-known closed-form expression for a 2-by-2 matrix inverse. We initialize the system in a steady-state mix of two and one fork states (Eqn. 4):

$$\begin{pmatrix} P_2(0) \\ P_1(0) \end{pmatrix} = \frac{1}{2k_- + k_+} \begin{pmatrix} k_+ \\ 2k_- \end{pmatrix}. \tag{13}$$

The average lifetime is therefore:

$$\bar{t} = \frac{4k_-^2 + 5k_-k_+ + k_+^2}{2k_-^2(2k_- + k_+)}, \tag{14}$$

which is measured in the replisome dynamics experiments.

Aside: Note that the independent-fork model does not appreciably affect the lifetime distribution function (Eqn. 8) for $R \approx 1$ relative to the cooperative model and therefore a new maximum likelihood fit need not be performed.

The underlying rates: Estimating the independent-fork model parameters. R is fit from the stoichiometry measurements and is therefore observable. We estimate the ratio is

$$R = 1.4 \pm 0.4 \tag{15}$$

from the focus stoichiometry measurements. We estimate the effective focus loss rate $k_{\rm eff}$:

$$k_{\rm eff} = 0.12 \pm 0.01 \text{ min}^{-1}, \tag{16}$$

from the replisome dynamics experiments.

Even though the ratio of one to zero fork replisomes cannot be directly measured, since non-replicating cells anomalously increase the number of zero-fork cells, we can still estimate this quantity in the model:

$$R_{10} \equiv N_1(\infty)/N_0(\infty) = 2k_+/k_- = 4R, \tag{17}$$

which can be expressed in terms of the observable ratio R. We therefore expect:

$$R_{10} = 5.8 \pm 1.6, \tag{18}$$

The probability of a conflict in a single fork is

$$P_c = \tfrac{k_-}{k_-+k_+} = \tfrac{1}{1+2R} = 0.26 \pm 0.05. \tag{19}$$

We now explicitly estimate the conflict and restart rates from the independent-fork model. The conflict rate per fork is:

$$k_- = k_{\rm eff}\frac{1+\frac{5}{2}R+R^2}{(1+R)} = 0.33\ \pm 0.09 \text{ min}^{-1}, \tag{20}$$

and the restart rate per stalled fork is:

$$k_+ = 2k_-R = 0.95 \pm 0.71 \text{ min}^{-1}. \tag{21}$$

We now estimate the number of conflicts per cell cycle. Assuming a replication time of T, we would expect:

$$N_c = k_-(1-P_c)T = \frac{k_-k_+}{k_-+k_+}T, \tag{22}$$

conflicts per fork per round of replication. Our naïve estimate for the cooperative model is:

$$N'_c \equiv k_{\rm eff}T. \tag{23}$$

The following table summarizes the conflict number predictions:

Replication time: T	Independent conflict number: N_c	Cooperative conflict number: N'_c
40 min	9.5 ± 0.8	4.8 ± 0.4
80 min	19.5 ± 1.6	9.7 ± 0.8
120 min	28.7 ± 2.4	14.5 ± 1.2

From the visualization of DnaN throughout the cell cycle, we estimate that the replication time under these growth conditions is 80-100 minutes (unpublished).

Discussion. It is important to note that both the cooperative and independent-fork models make a number of simplifying assumptions. For instance, these models do not include the known position of conflicts at the rDNA and other loci. In the model, we treat the generation of conflicts as a Poisson process with a constant rate. The existence of these sequence-specific hot spots could act to over-estimate the rate of conflicts if these forks undergo multiple rounds of abortive restart. Our dynamics experiments also do not detect short-lived conflicts that are resolved quickly. The independent-fork model also assumes that there is no interaction between the forks. Our data suggest that the forks are frequently co-localized, which is consistent with a role for functional coupling between forks.
